# Volume One

**Published:** 1881-12

**Authors:** 


### HOMŒOPATHIC PHYSICIAN.

A MONTHLY JOURNAL OF MEDICAL SCIENCE.

"If our school ever gives up the strict inductive method of Hahnemann, we are lost, and deserve to be mentioned only as a caricature, in the history of medicine."—CONSTANTINE HERING.

Vol. I. DECEMBER, 1881. No. 12.

### EDITORIAL.

VOLUME ONE.—In closing our first volume we can not forbear making a few rambling comments, as to the past and the future. Our object, in the past, has been to teach, as best we could, *pure* homœopathy, to illustrate it practically, to bring out the characteristics of old remedies, to introduce new—in short to do such work as would make us all more efficient in healing the sick. Thanks to the gentlemen who have contributed to our pages, we have been enabled to make a good start in this work. Thanks to our large (and steadily increasing) circulation, we have been able to publish over *two hundred* pages more than we promised in our prospectus. If we have done well in the past, we shall strive to do even better in the future.

*In the past*, we have endeavored to point out some of the pernicious innovations that are being introduced into homœopathic practice; to condemn such errors as the use of unproved remedies, the alternation or combination of remedies, the use of palliatives and tonics, etc.

*In the future*, we shall continue to teach the Homœopathy of Hahnemann; shall try to enrich our materia medica and hope to give practical articles on the treatment of various diseases.

The periscope will contain, in a condensed and available form, all that is practically useful in current homœopathic literature. In our book notices, reviews, etc., we will keep our readers informed as to all the best and newest works on medicine.

